# Adolescent experiences of pregnancy in low-and middle-income countries: a meta-synthesis of qualitative studies

**DOI:** 10.1186/s12884-022-05022-1

**Published:** 2022-09-12

**Authors:** Rachel Crooks, Carol Bedwell, Tina Lavender

**Affiliations:** 1 Liverpool School of Tropical Medicine, Liverpool, UK; 2 Department of International Public Health, Liverpool School of Tropical Medicine, Liverpool, UK

**Keywords:** Meta-synthesis, Adolescent, Pregnancy, Low-and middle-income countries

## Abstract

**Background:**

Fertility rates among adolescents have fallen globally, yet the greatest incidence remains in low-and middle-income countries (LMICs). Gaining insight into adolescents needs and experiences of pregnancy will help identify if context specific services meet their needs and how to optimise pregnancy experiences. A meta-synthesis of qualitative studies considering adolescent experiences of pregnancy in LMICs has not yet been published.

**Aim:**

To synthesise available qualitative evidence to provide greater understanding of the needs and experiences of adolescents who become pregnant in low-and middle-income countries.

**Methods:**

An extensive search utilised six databases and citations searching. Studies were included if they were of a qualitative or mixed methods design. Participants lived in LMICs and were adolescents who were pregnant, had experienced pregnancy during adolescence or were an adolescent male partner. Relevant studies were assessed for quality to determine suitability for inclusion. A meta-ethnography approach was used to generate themes and a final line of argument.

**Results:**

After screening and quality assessment 21 studies were included. The meta-ethnography generated four themes, *A wealth of emotions*, *I am not ready*, *Impactful relationships* and *Respectful and disrespectful care*. Unplanned, unwanted and unacceptable pregnancies were a source of shame, with subsequent challenging personal relationships and frequently a lack of needed support. Even when pregnancy was wanted, adolescents faced the internal conflict of their desires not always aligning with socio-cultural, religious and family expectations. Access, utilisation and experiences of care were significantly impacted by adolescents’ relationships with others, the level of respectful care experienced, and engagement with adolescent friendly services.

**Conclusions:**

Adolescents who experience pregnancy in LMICs deserve support to meet their personal and pregnancy needs; efforts are needed to tailor the support provided. A lack of a health care provider knowledge and skills is an obstacle to optimal support, with more and better training integral to increasing the availability of adolescent friendly and respectful care. Adolescents should be involved in the planning of health care services and supported to make decisions about their care. The diversity across countries mean policy makers and other stakeholders need to consider how these implications can be realised in each context.

**Supplementary Information:**

The online version contains supplementary material available at 10.1186/s12884-022-05022-1.

## Background

Despite falling adolescent fertility rates globally, births to adolescents still account for 11% of total annual births worldwide [1]. The greatest incidence is in LMICs, with the adolescent birth rate in the world’s poorest countries four times higher than in high-income regions [2–4]. Pregnancy in adolescence is more common in Latin America and the Caribbean, South Asia and sub-Saharan Africa than anywhere else in the world [5, 6], with 29 of the 34 countries reporting adolescent fertility at more than 80 per 1000 between 2015–2020 to older adolescent girls, in Africa [7].

Globally, adolescents who are poor, rural living and less educated are most likely to become pregnant, as they have less power, opportunities and choices than their peers [8–10]. In LMICs approximately half of pregnancies are unintended [11], with peer pressure, substance misuse and the media identified as factors influencing adolescents sexual risk-taking behaviour and experiences of becoming pregnant [12, 13]. Whilst sexual risk-taking is not isolated to adolescents in these regions, gender power imbalances with an associated need to placate partners, sexual coercion and transactional sex increase adolescent’s vulnerability to unintended pregnancy [12–15].

The high pregnancy incidence in the identified regions is associated with a significant unmet need for contraception; evidenced by countries in sub-Saharan Africa with the highest adolescent birth rates also reporting the lowest use of modern contraceptives [6, 11]. Adolescent’s engagement with sexual and reproductive health services is influenced by a lack of pregnancy and prevention of pregnancy knowledge, myths about the harms of contraception, poor health worker attitudes and legislation restricting contraception to married women [8, 15, 16]. Fear among adolescents of social stigma associated with pre-marital sex and concern they will be perceived as promiscuous or being unfaithful to their partners, are additional deterrents for adolescents accessing sexual and reproductive health services or using contraception [8, 11, 17].

The low incidence of pre-marital sex and unintended pregnancy across Asia [18] suggests pregnancies are more likely to be planned and occur within marriage. Pregnancies occurring within marriages may be intended, but are often not freely chosen by adolescents, with social pressures to conceive, coercion from family members and a lack of control over contraceptive choice and use, enabling pregnancy in adolescence [3, 19–21]. Adolescents living in contexts with socio-cultural norms, such as beliefs the only role for girls is to bear children, that pregnancy marks the transition to womanhood and is proof of maturity, and that pregnancy is a means of gaining respect within society, also face a predisposition to pregnancy in adolescence [6, 7, 22, 23].

The consequences of pregnancy in adolescence are well documented, with adolescents’ life trajectories altered when they become pregnant, propelling them prematurely into adulthood, with opportunities for education, employment and associated freedoms, opportunities and choices all reduced [18]. Psychosocial and economic consequences are particularly challenging amongst unmarried adolescents who can experience stigma, social isolation and being ostracised from their families with ongoing adverse psycho-social implications [24, 25].

Pregnancy complications and unsafe abortions are the leading cause of death amongst older adolescents [26] and a higher incidence of childbirth related morbidities, such as obstetric fistula, are seen in young adolescents compared to older adolescents and adults [4, 27]. Adolescents and their babies face an increased risk of pregnancy and childbirth related mortality and morbidity compared to adult women [28–32]. Although these outcomes are undeniably associated with physiological age, other factors including reduced care-seeking and insufficient antenatal care among adolescents are also associated with an increased risk of complications [33–35].

Despite efforts to reduce the incidence of pregnancy in adolescence, including preventing child marriage, keeping girls in education and improving access to contraception [36], adolescents continue to become pregnant and few LMICs adequately prioritise their care [4, 37]. Gaining insight into adolescents’ experiences of pregnancy will help identify if context specific services meet their needs, if they require additional support, and how to optimise their pregnancy experience. Several researchers have conducted studies exploring adolescent pregnancy experiences in LMICs [38–41], but a meta-synthesis will produce an integrative interpretation, more substantial than can be afforded by individual studies [42, 43].

Despite the increasing interest in the value of synthesising qualitative studies, to inform future research, policy and practice [42, 43], a meta-synthesis of qualitative studies considering adolescent experiences of pregnancy in LMICs has not yet been published. Meta-syntheses have focused on pregnancy in adolescence [44–46], but most of the included studies were undertaken in high-income contexts. The significant contextual differences mean findings are unlikely to be transferrable to LMICs which, considering the increased incidence in these regions, highlights an important area of research warranting further exploration.

## Main text

### Aim

This review aimed to synthesise available qualitative evidence to provide greater understanding of the needs and experiences of adolescents who become pregnant in low-and middle-income countries.

## Methods

The meta-ethnographic approach, which focuses on interpreting what a collection of studies can contribute as a whole [47], was used for the synthesis. This review was registered with PROSPERO, the International prospective register for systematic reviews (PROSPERO ID: CRD42021251591).

### Inclusion criteria

Mixed methods or qualitative empirical studies of any methodology were included. Participants were male or female and adolescents, aged 10-19 years, in accordance with the definition provided by the World Health Organization [26], who were pregnant, had experienced pregnancy during adolescence or were an adolescent male partner. The location of the study had to be a low or middle income country, as classified by The World Bank [48]. Only English language papers were included, and no date restrictions were applied.

### Search strategy

A search strategy was developed, based on an initial scoping search, to identify papers relevant to the review aims. Search terms were formulated using the SPIDER search strategy tool [49], designed for use in qualitative research (Table 1). Searches of the databases Cumulative Index of Nursing and Allied Health Literature (CINAHL Complete), Medline complete, Global Health, PubMed and African Journals Online and PsycINFO, were conducted in May 2021 and repeated in February 2022. As poor indexing of qualitative studies can mean database searches fail to yield optimal results [50, 51], papers suitable for inclusion were also citation searched. Search terms specific to each database can be seen in the supplementary information (S1).

**Table 1:** Initial search strategy using the SPIDER search strategy tool

Spider Tool	Search Terms (Keywords)
S—Sample	S1: Tx Adolescen* OR teen* OR young* S2: Tx “Low-and middle-income countries” OR “Developing Countries” OR LMIC
PI—Phenomenon of Interest	S3: Tx Pregnan* OR antenatal OR prenatal
D—Design	S4: Tx “Focus Groups” OR interview* OR “case stud”*
E—Evaluation	S5: Tx Experience* OR attitude* OR feel* OR thought* OR view* OR perspe* OR encounter* OR opinion* OR belief* OR perce* OR understand* OR expect*
R—Research Type	S6: Tx Qualitative OR “mixed methods”
Combining search terms: S7: S1 AND S2 AND S3 AND S5 S8: S4 AND S6 S9: S7 AND S8	

### Quality appraisal

A quality assessment of included studies was undertaken, as the quality of included studies has been found to impact the trustworthiness of review findings [52–54], using the checklist tool developed by Walsh and Downe [53] (Supplementary Information – S2) and the grading categories described by Downe, Simpson and Trafford [55] (Table 2). Consistent with the approach of other authors who utilized these tools [56–58], studies graded C or above were considered of sufficient quality for inclusion, with those graded D excluded from the synthesis.

**Table 2:** Quality assessment grading as described by Downe, Simpson and Trafford [55]

**A—No or few flaws**	High credibility, transferability, dependability and confirmability
**B—Some flaws**	Unlikely to affect the credibility, transferability, dependability and/or confirmability of the study
**C—Some flaws**	May affect the credibility, transferability, dependability and/or confirmability of the study
**D – Significant flaws**	Very likely to affect the credibility, transferability, dependability and/or confirmability of the study

### Data extraction and synthesis

The meta-ethnography approach, involves analysing the primary studies to appreciate their collective meaning, described as discovering a whole from all of the parts, to generate a final line of argument [47, 59, 60]. This was achieved by using a constant comparative method [61] to consider how the studies were related, looking first for similarities (reciprocal findings) between the papers and then by looking for conflicts with the evolving concepts (refutational analysis). The process was iterative, with emerging themes revised, until final themes were drawn together to form a line of argument.

To enhance the trustworthiness of the synthesis and reduce bias, an audit trail was maintained, multiple authors were involved in the review process and reflexivity was maintained throughout. Authors met regularly to discuss the review and acknowledge how our positionality, as midwives with varied experiences of working with adolescents in low-and middle-income countries, may have impacted assumptions and interpretations.

## Results

**Fig. 1:**
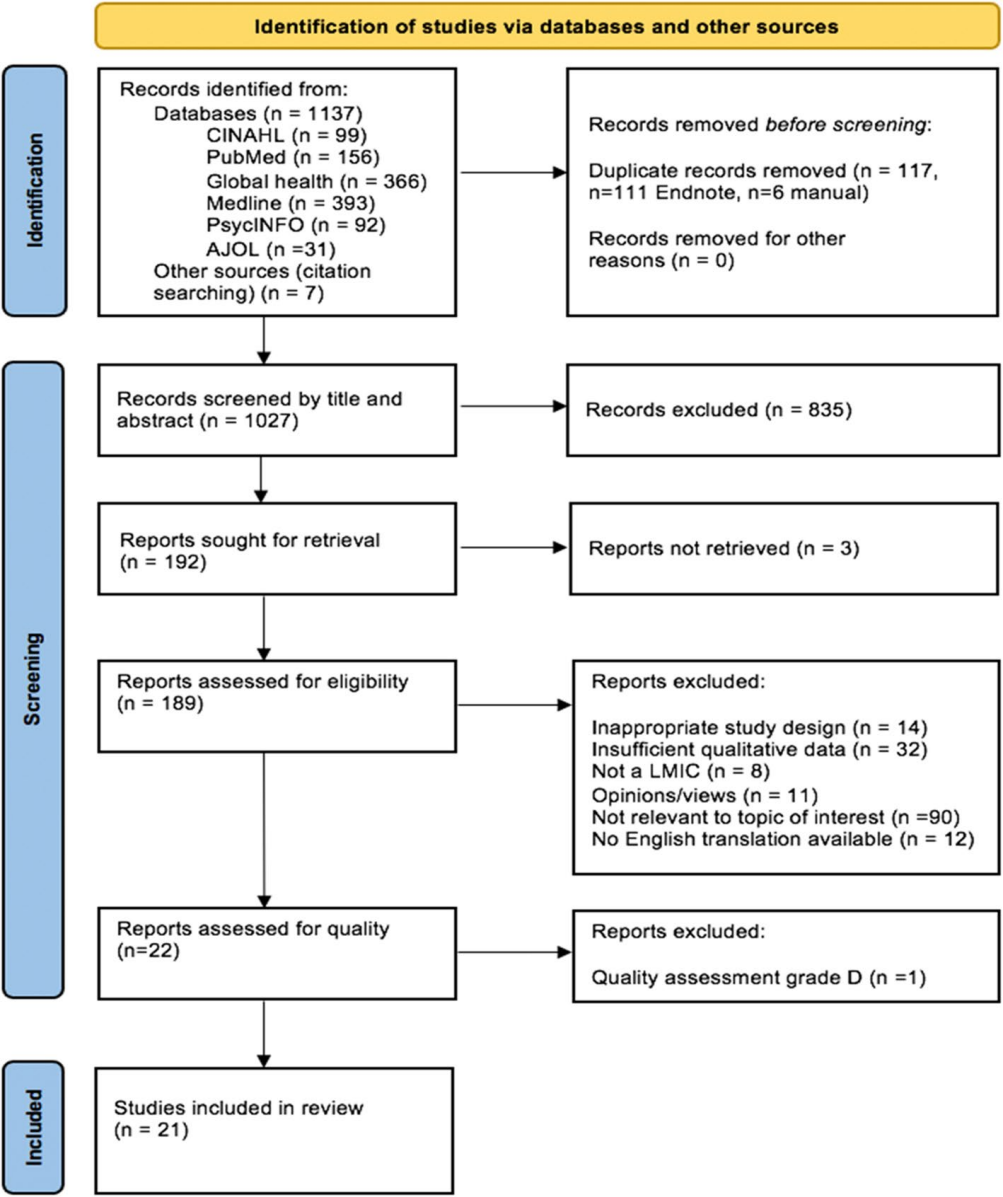
Prisma flow diagram [65] showing process of identifying studies for inclusion

The search strategy generated 1,144 papers, 1,137 from database searches and seven from citation searching. The search in African Journals Online generated 954 results, however only the first 100, classified as the most relevant papers, were accessible, which is a limitation of this review. After removal of 117 duplicates, 1027 papers were screened by reading the title and abstract which lead to 835 exclusions. Full texts were sought for the remaining 192 papers, however, three were not found despite contacting the authors directly. One hundred and eighty-nine full texts were read, with 167 studies excluded, for one of six reasons detailed in Fig. 1. The remaining 22 studies were quality assessed, with 25% of the papers reviewed by the other two authors to ensure consistency and agreement on grading. Twenty-one papers were graded ‘C’ or above and were included in the review. Studies graded ‘C’ lacked detail in some of the components assessed, rather than obvious methodological flaws which may affect the trustworthiness of findings. For example, two studies used appropriate methods and analysis but lacked detail on sampling strategies [62, 63]. One paper [64] was excluded with a grade of ‘D’ because few of the components assessed were identified in the paper, with poor reporting of the methods and results. A summary of the quality assessment can be found in the supplementary information (S3). Study characteristics are summarised in Table 3 (below).

**Table 3:** Characteristics of included studies

Identifier (author/year) (citation)	Country	Aim	Design	Sample & recruitment	Data collection methods	Method of analysis	Quality grade
Al-Kloub et al. (2019) [38]	Jordan (MENA)	To understand the lived experience of marriage and motherhood among Jordanian young women with a first-born child To identify needs of Jordanian adolescent mothers and to develop the support necessary to enhance their maternal role and health outcomes	Descriptive qualitative	15 participants 10–14 years = 2 15–19 years = 13 All married Hospital birth registers used to identify potential participants and snowball sampling through hospital recruited participants	Interviews	Colaizzis method	B
Apolet et al. (2020) [62]	Uganda (SSA)	To explore the challenges faced by adolescents during the antenatal, delivery and postnatal period and the extent to which the community score card intervention in Kibuku district addressed those challenges	Qualitative	15 participants No breakdown of ages Mix of married and single Sub-county score card coordinators and village health team (volunteers actively involved in coordinating health related issues at community level) identified adolescents who had given birth in previous two years	IDIs	Manual thematic framework analysis	C
Asnong et al. (2018) [66]	Thailand (SEA)	To develop a better understanding of adolescent pregnancy, including sexual and reproductive health knowledge and family and community support structures on the Thailand-Myanmar border	Qualitative	20 participants (others key informants) 10–14 years = 2 15–19 years = 18 All married 76 potential participants identified from a clinic using convenience sampling. Participants were selected as they were attending for ANC during the study period	IDIs	Thematic analysis	B
Astuti et al. (2020) [67]	Indonesia (SEA)	To explore female and male Indonesian adolescents’ experiences during pregnancy and early parenthood, because of premarital pregnancy	Explorative qualitative approach	20 participants 10–14 years = 0 15–19 years = 20 (16–19) Married/due to because pre-marital conception All potential participants were identified from antenatal care records or postnatal records	Interviews	Colaizzi method	B
Atuyambe et al. (2005) [63]	Uganda (SSA)	To explore problems experienced by pregnant adolescents to design appropriate policies and programs, and to raise issues for further research	Explorative qualitative design	44 participants Age breakdown not specified Marital status not specified Participants selected from the community and among those receiving ANC at health units	FGDs	Manual Analysis—no further detail provided	C
Atuyambe et al. (2009) [68]	Uganda (SSA)	To explore adolescent health seeking behaviour during pregnancy and early motherhood to contribute to health policy formulation and improved access to health care	Qualitative	92 participants (others key informants) 10–14 years = 0 15–19 years = 92 Married and single Recruited at antenatal or vaccination clinic	FGDs	Latent content analysis technique used	B
Bwalya et al. (2018) [39]	Zambia (SSA)	To describe the experiences of pregnant adolescents with the healthcare providers at the antenatal care clinic To describe the experiences of pregnant adolescents with older pregnant women within the antenatal care clinic To describe the experiences of pregnant adolescents with education provided at as part of antenatal care	Phenomenological study	Sample size of 16 but only 12 participants interviewed as reached saturation 10–14 years = 0 15–19 years = 12 Purposively selected from antenatal clinic records	SSIs	Thematic analysis	C
Chikalipo et al. (2018) [40]	Malawi (SSA)	To explore the views of pregnant adolescents towards the antenatal services they receive at Ndirande Health Centre in Blantyre, Malawi, specifically their perceptions of the care received	Exploratory study	15 participants Age breakdown not provided. All 14–19 years Married and single participants Purposively selected at antenatal clinic when attending for care	SSIs	Manual thematic analysis	B
Duggan and Adejumo (2012) [69]	South Africa (SSA)	To determine how well the existing maternity services catered for the needs of adolescent maternity clients (AMCs)	Grounded theory	18 participants 10–14 years = 0 15–19 years = 18 Purposive and snowball sampling. Health care workers working in facilities within study area assisted with identifying potential participants	FGDs and SSIs	Manual thematic analysis	A
Erasmus, Knight and Dutton (2020) [70]	South Africa (SSA)	To explore the perceptions and experiences of pregnant adolescents utilizing one Midwifery Obstetric Unit (MOU) in urban Western Cape for their maternal health needs, to understand and explore barriers to access to care amongst pregnant adolescents within this specific context	Exploratory qualitative design	10 participants (others key informants) 10–14 years = 0 15–19 years = 10 Married and single participants Purposive sampling. No detail of recruitment process	SSIs	Manual thematic analysis	B
Govender, Naidoo and Taylor (2020) [41]	South Africa (SSA)	To explore and understand the phenomenon of adolescent pregnancy and motherhood To gain insight into the future aspirations of adolescent mothers	Descriptive	18 participants 10–14 years = 0 15–19 years = 18 Participants single or in relationships, not married Purposively selected from quantitative strand, as part of larger mix-ed methods study	FGDs	Thematic analysis	B
Gyesaw and Ankomah (2013) [23]	Ghana (SSA)	To explore the experiences of unmarried teenage mothers in relation to pregnancy, delivery, and early motherhood	Qualitative	63 participants No breakdown. All participants 14–19 years. All single Health professionals assisted with recruitment. Snowballing also used through those adolescents identified at facilities. Key persons in the community also helped identify eligible adolescents	FGDs and IDIs	Manual thematic analysis	B
James, Rall and Strümpher (2012) [71]	South Africa (SSA)	To explore and describe the perceptions of pregnant teenagers of the ANC clinic environment To recommend guidelines to midwifery operational managers for strategies to create teenager-friendly ANC clinic environments	Qualitative	12 participants No age ranges provided Potential participants identified from antenatal clinic register	SSIs	Transcription and analysis withing 12 h	C
Mashala et al. (2012) [72]	South Africa (SSA)	The aim of the study was to explore and describe the experiences and challenges of pregnant South African adolescents by focusing on their thoughts and feelings about their pregnancies and prospects	Phenomenological study	9 participants No breakdown of ages (Mean age = 16.33 years) No recruitment processes explained. Likely purposively sampled at health facility—not clear	SSIs	Thematic analysis	C
Mohammadi et al. (2016) [73]	Iran (MENA)	To explore the lived experiences of pregnant teenage women in Iran	Hermeneutic phenomenology	11 participants 10–14 years = 0 15–19 years = 11 – All married Participants recruited from health facilities where they had registered for antenatal care	SSIs and IDIs	Thematic analysis	B
Nabugoomu et al. (2018) [74]	Uganda (SSA)	To understand community stakeholder perceptions of the needs of teenage mothers in rural Eastern Uganda To understand the barriers, they face in meeting those needs	Qualitative	11 pregnant adolescents, 14 lactating adolescents = 25 (others key informants) Ages of adolescents not specified Recruited through community health team workers	SSIs	Thematic analysis	B
November and Sandall (2018) [75]	Sierra Leone (SSA)	To better understand the factors which put younger women at greater risk of maternal death, to work with local people to develop and evaluate interventions to reduce these risks	Qualitative	20 adolescent participants (others key informants) No age ranges specified Recruited within a local training institution or through community health workers	FGDs and SSIs	Software used to aid coding and analysis- Doesn't state type of analysis	C
Sewpaul et al. (2021) [76]	South Africa (SSA)	To investigate pregnant adolescents' general knowledge, attitudes, motivating factors, and experiences of antenatal appointment attendance and their healthcare behaviours during pregnancy	Phenomenological study	19 participants aged 13–19 years. No breakdown of ages or marital status. Purposive sampling, with participants recruited at health facilities	FGDs and IDIs	Thematic analysis	B
Shahabuddin et al. (2017) [77]	Bangladesh (South Asia)	To explore maternal health care-seeking behaviour of adolescent girls and their experiences related to pregnancy and delivery in Bangladesh	Prospective qualitative study	25 adolescent participants and 23 at follow up. (Other key informants). No ages provided. All married Purposive sampling with support of field staff working on maternal health project	FGDs and IDIs	Thematic analysis	B
Shahabuddin et al. (2019) [78]	Nepal (South Asia)	To explore the health care-seeking behaviour of married adolescent girls in Nepal during pregnancy, delivery and post-delivery	prospective qualitative study	22 participants and 18 at follow up. (Others key informants) No ages provided for pregnant adolescents. All married Purposive sampling with support of field staff working on maternal health project	IDIs	Thematic analysis- guided by the Social-Ecological Model (SEM)	B
Tatum et al. (2012) [79]	Mexico (LAC)	To examine the factors influencing how young women in a setting in which abortion was recently legalized make reproductive decisions when confronted with an unwanted pregnancy	Qualitative	23 participants 10–14 years = 5 15–19 years = 18 Recruitment for IDIs and FGDs was conducted by private recruiters. The recruiters, who maintain a database of contacts acquired through snowball sampling, used these contacts to locate suitable participants	FGDs and IDIs	Thematic analysis	C

### Description of themes

Emerging themes evolved throughout the synthesis process, with final themes and associated core concepts generated, as summarised in Table 4, which contributed to the final line of argument.

**Table 4:** Evolving themes and core concepts

Evolving themes	Relevant studies	Final themes	Core concept
**Psychological and social factors** Stigma Suicidal attempts/ideation Mixed emotions Fear of reactions to pregnancies Unprepared for birth Not ready for motherhood	[23, 38, 38–41, 62, 63, 66–68, 68–70, 72, 73, 73, 74, 76, 78, 79]	**A wealth of emotions** Reacting to pregnancy Mixed emotions	Socio-cultural and religious expectations increase the stigma experienced by adolescents
		**I am not ready** Too young to be a mother Unprepared for birth	Not adequately prepared for birth or ready for motherhood
**Relationships** Family Peers Partners Religion	[23, 62, 63, 66, 66–70, 70, 72, 72, 74, 75, 77–79]	**Impactful relationships** Acceptance, support and encouragement Rejection, harm and abandonment	Relationships with others have a direct and significant impact on adolescents needs and pregnancy experiences
**Health care services** Lack of adolescent friendly services Health worker attitudes Older women	[39, 39, 40, 40, 41, 62, 62, 63, 68–71, 71, 76, 76–78]	**Respectful and Disrespectful Care** Health workers Older women	Lack of adolescent friendly and respectful provision of health care

### A wealth of emotions

#### Reacting to pregnancy

Consistent among the studies which addressed discovery of a pregnancy were the fraught emotions and psychological challenges adolescent’s experienced [41, 67, 70, 72, 79]. Feelings of fear, anger, guilt and shame were associated with the disappointment of no longer meeting religious, socio-cultural or parental expectations that unmarried adolescents should refrain from sexual relationships [41, 67, 70, 72, 79], as evidenced:

*“I had intense feelings of guilt and shame. I had previously attended the reed dance and I was so proud of my virginity”* [41].

The only study that actively recruited male adolescents noted a difference in the reactions of male and female participants [67]. Female participants were fearful, and in some instances, desperate to end the pregnancy. Whereas most male adolescents were open to the pregnancy, seemingly compelled by religious conviction:

“…. I didn’t want to kill my baby…I was the kind of person who did a sin and was adding more and more sin…” [67]

Profound psychological implications of this distress were evident in participant reports of suicidal ideation and attempts [41, 67, 79]:

*“…I suspected that I might be pregnant, I attempted suicide, but I failed…I took a pregnancy test. The test was positive. I attempted suicide again…”* [41].

Whereas married participants in the Middle East and North Africa [38, 73] expressed conflicted feelings on discovering their pregnancies, suggesting a sense of ambivalence to pregnancy, as evidenced:

*“I was happy getting pregnant because it made me a mother, but at the same time I believe being unmarried is better…I liked pregnancy and did not like it at the same time…”* [38].

#### Mixed emotions

Emotions and feelings experienced by adolescents as the pregnancy continued were more varied. Continued negative feelings associated with shame, fear and isolation were evident in two studies from sub-Saharan Africa [41, 68], for example:

*“My repeat pregnancy has made life very challenging. I feel secluded. I don’t have a social life.*” [41].

However, participants were identified in three studies that shared a sense of purpose, happiness and thankfulness on seeing their babies [23, 38, 73], as reflected in the quote below. This suggests becoming a mother can be a positive experience, even if pregnancy is not.

*“…But when I delivered the baby and saw him all my feelings changed. I love him so much…”* [38].

### I am not ready

#### Too young to be a mother

Not being ready for pregnancy, childbirth or motherhood, was suggested in several studies [38, 68, 73, 78]. Feelings and experiences of lost childhood, early development and not being emotionally ready to transition from being mothered to mothering, were seen in the studies of married adolescents in the Middle East and North Africa [38, 73]:

*“I still like to be with my mother. I need to be loved by my mom… It often keeps my mind busy that I’m not ready emotionally…”* [73].

There were few accounts from participants of desiring or choosing pregnancy in this life stage [72, 78], with the majority of married participants in Nepal [78] acknowledging little decision-making power over their pregnancy choices. However, some participants did:

*“No, I didn’t consult with anyone. I was willing to have a child, so I consulted with my husband and decided on having only one child…”* [78].

#### Unprepared for birth

Adolescents were often psychologically, emotionally and practically ill prepared for labour and childbirth [38, 40, 62, 66, 74, 76]. A lack of mental preparedness led to fearful and negative birth experiences [38–40], as evidenced:

*“I had no idea about the birth process except…How the baby came out I did not know, I did not imagine the severity of the pain…”* [38].

Adolescents wanted, appreciated and benefitted from antenatal education, when it was available, but there was evidence of a lack of discussion and information about labour and childbirth [39, 40, 76]:

*“They needed to tell us what we should expect during labour, we are young we know nothing…so we are afraid”* [40].

Four studies in sub-Saharan Africa described adolescent’s awareness of the materials required for birth and immediate care of the baby [40, 62, 74, 76], but recognised inability to practically prepare was due to a lack of financial means [62, 74], for example:

*“The midwife gave us a list of items to buy for delivery…but I do not have any of them…even getting money for buying medicines and food is a problem…”* [74].

### Impactful relationships

#### Support, acceptance and encouragement

The need for, and benefit of support, acceptance and encouragement was inferred in several studies [41, 66, 67, 70, 72, 79]. Fathers were more dismissive or took longer to accept the pregnancy than Mothers [23, 70, 72, 75]. While support from women in the family was particularly helpful, with mothers, sisters and grandmothers having a caring role [66, 70, 72], for example:

*“My ma [grandmother] was disappointed [about the pregnancy] but ...she said that…she will stand by me”* [70].

Participants also wanted and benefitted from having a birth partner during labour and childbirth [69, 77, 78]. Both studies in South Asia reported the desire for a female birth companion during labour was one of the reasons for choosing home delivery over hospital birth [77, 78]. Many participants desired and appreciated comfort, encouragement and support, as evidenced:

*‘‘I wouldn’t have made it without my mother’’* [69].

#### Shame, rejection and abandonment

Many participants, with unplanned pregnancies, shared fears of families finding out about the pregnancy, bringing shame on the family, parental reactions and subsequent serious adverse consequences [63, 70, 72, 79], for example:

*"… I have a very harsh father. I fear that if I tell him he can beat me up… at times when you are not willing to leave home, he sends you away"* [63].

Pregnancy caused strained and damaged family relationships for many participants, particularly with parents [23, 62, 63, 66, 70, 72, 74, 75], with widely reported experiences of neglect, physical violence and being forced to leave the family home, for example:

*“My parents do not trust me anymore. They abandoned and treated me badly, abusing and chasing me away...”* [74]

Denial, rejection and abandonment by partners, also had significant implications, with participants reporting unmet emotional, financial and practical needs [23, 62, 68, 72, 74]:

*"The father of this child after making me pregnant denied it. So… I started living with my grandmother….the baby’s father lives in same village but does not give any support”* [68].

### Respectful and disrespectful care

#### Health care professionals

Fear of health care providers poor treatment was a deterrent to accessing health care services [62, 63, 68]. While reports of health care providers’ poor attitudes and behaviours, such as having a rude manner, being judgemental or discriminatory, and physical violence and aggression, directly contributed to negative experiences [40, 62, 63, 68–71, 76], for example:

*“The nurses treated me badly during my pregnancy. They embarrassed me at my first antenatal visit. The doctors can also be judgmental….”* [41].

Health care provider prejudices against adolescents, particularly those that were unmarried, meant some participants were denied or experienced delayed care [39, 62, 71, 76]:

*“…they give first priority to those women who come with their husbands… even when they come late for antenatal care”* [39].

Interactions with health care providers who were, kind, gentle and friendly had a positive impact on adolescent’s experiences [39, 40, 69, 70, 76], with some participants pleasantly surprised by their positive experiences:

*“There's some nurses that's nice to you and show you respect and they always helpful, talk to you, ask you questions …Treat you with love and respect…”* [76]

Husbands and Mothers-in-law had the greatest impact on care seeking and care utilisation, among married adolescents [38, 77, 78]. However, not wanting to be cared for by male doctors, because of religious prohibitions [77] or shyness [78] was also a deterrent:

*“…I heard male doctor will be in a medical (hospital or clinic) it makes me feeling shy!”* [77]

#### Older women

Positive or negative experiences of health care services were also influenced by adolescent’s interactions with adult women [39, 40, 62, 71, 76]. Being shamed or intimidated by older women [39, 62, 71, 76], prevented adolescents from accessing care services and resulted in negative experiences when they did, as evidenced.

*“I overheard some women talking in a mocking manner…This made me to feel uncomfortable and ashamed of myself. They were even laughing at me”* [39].

The value and desire for adolescent specific services was reflected in two studies, with participants sharing the benefits of being able to speak and interact freely without feeling constrained by older women [40, 62]. However, most participants in the study in Zambia [39] reported healthy relationships with older women, acknowledging them to be friendly, supportive and a source of guidance:

*“l ask them pregnancy related questions… They teach me on how to take care of my pregnancy…They teach me things l don’t know”* [39].

### Line of argument

The myriad of emotions and profound psychological implications for adolescents who experienced pregnancy in LMICs was seemingly driven by socio-cultural and religious expectations. Unplanned, unwanted and unacceptable pregnancies were a source of shame, with subsequent challenging personal relationships and all too often a lack of needed support. Even when pregnancy in adolescence was wanted, planned, and acceptable within communities, adolescents faced the internal conflict of their desires not always aligning with socio-cultural, religious and family expectations. Other peoples’ responses and actions significantly contributed to adolescent’s experiences, mental and practical preparedness and empowerment to make decisions about their personal and pregnancy needs. Access, utilisation and experiences of care were also significantly impacted by adolescents’ relationships with others, with negative experiences overwhelmingly associated with a lack of respectful and adolescent friendly services.

## Discussion

This review aimed to analyse, interpret and synthesise qualitative studies to provide a current comprehensive understanding of the needs and experiences of adolescents who become pregnant in LMICs. The described themes and associated core concepts contributed to the subsequent line of argument.

There were notable differences in how adolescents felt about their pregnancies between married and unmarried adolescents and between culturally diverse regions. The predominantly negative emotions experienced by unmarried adolescents [62, 67, 70] reflected the influence of religion, culture and societal norms on perceptions, experiences and behaviours cited in wider literature [80–82]. Ambivalence to religious prohibitions on premarital sex has been suggested to decrease the suicide risk among pregnant adolescents [83]. However, in countries where culture and religion have such a distinct influence and control over lived experiences, ambivalence to these expectations is less likely than in high-income countries (HICs) where individualism, autonomy and choice are acceptable and encouraged [84].

The concept of not being ready for motherhood was most strongly reflected among married adolescents. Although adult women in these regions experience the socio-cultural norms and expectations to bear children [82, 85, 86], the sentiments of lost childhood, developing too quickly and not being ready for motherhood [38, 68, 73, 78], identified in this review, are age specific. Similar sentiments were identified among adolescents with planned and unplanned pregnancies in HICs [45, 46, 87], but were not distinctly associated with marital status. The findings of this review support the concern expressed by other authors that planned pregnancies are not always chosen by adolescents or wanted by them in this life stage [3, 82] and that global efforts to prevent unwanted pregnancies in adolescence should remain a priority.

Feeling unprepared and fearful of childbirth was identified in both married and unmarried adolescents. Likely due to a societal hesitancy, associated with socio-cultural and religious beliefs, which discourage and prevent discussions about sexual and reproductive health with adolescents, as well as reluctance from adolescents to engage in these conversations [5, 15, 88]. Feelings of being unprepared, linked to a lack of financial means to purchase the materials required for birth, were only reported in the low-income countries of Malawi [40] and Uganda [62, 74]. Studies in high-income regions have identified financial challenges for pregnant adolescents but note the benefits of welfare support [45, 46, 89] which, while available in some LMICs, is less likely to be available in low-resource contexts. There is a need for states and other stakeholders to consider how pregnant adolescents in low-income countries can be better supported to meet their daily and pregnancy related needs.

The need for support and nurturing is expected of individuals in this life-stage [90, 91], yet this review found an important unmet need for love, care and support in personal relationships. Findings that adolescents feared or experienced family and partner harm, rejection, and abandonment, with the associated short and long-term physical, psycho-social and economic consequences [23, 62, 68, 72, 74], are consistent with outcomes for adolescents described in wider literature [24, 25, 37]. Reports from adolescents experiencing pregnancy in other contexts, that being loved, supported and encouraged, helped them to manage their pregnancy, have some positive pregnancy experiences and feel more prepared for motherhood [45, 46], speak to the benefit of addressing this unmet need.

Even when adolescents were cared for and supported within a family unit, this review recognised a lack of autonomy and decision-making. Adolescents need to be empowered to make whatever choices they can. Women’s lack of agency in decision-making and access to care is not exclusive to adolescents, with Mothers-in-law and Husbands commonly reported as the decision-makers in South Asia, the Middle East and North Africa, and parts of sub-Saharan Africa [92–94]. Engaging families in measures to improve care provision could increase care-seeking among adolescents and enhance the family’s role as a source of support. Education is a key aspect of empowerment, giving individuals control of their reproductive health [95]. Adolescent focussed health care provision, with supportive health workers, may help to empower pregnant adolescents through education, and support for choices made. A reluctance to access services was also identified, either for religious reasons [77] or because of shyness, embarrassment, and an increasing desire for privacy [5, 78]. This review supports the recommendation that care must be attentive to the religious, cultural and developmental needs of service users [80]. Accommodating this need for privacy is an essential component of care provision and could improve care-seeking.

Disrespectful attitudes and behaviours of health care providers and other service users, were all reported from sub-Saharan Africa [39, 62, 63, 68–71, 76], which reflects the findings of other authors that disrespectful care remains a considerable challenge in this region [96]. The experiences of disrespectful care identified are consistent with reports from older women [97–100]. As well as positive interactions with health care providers, being merely the absence of harmful attitudes and behaviours [101, 102]. However, this review found age specific experiences including adolescents being treated differently to adult women and experiencing poor treatment from older women [39, 62, 71, 76], that suggest they face a double burden of discrimination related to both gender and age. Poor treatment of adolescents has been acknowledged by health care providers, citing a lack of knowledge and training as reasons for this behaviour [94, 102, 103]. Yet the importance of positive attitudes, knowledge and skills of health care providers to engage with adolescents is widely acknowledged [5, 104–106]. This review contributes additional evidence to this body of research, supporting a need for more training to enable health care providers to meet the support needs of adolescents who are pregnant in LMICs.

Findings suggest adolescents’ experiences of health care were more positive when services were tailored to accommodate their needs and preferences [40, 62] and that adolescents can and would like to influence the care they receive [39, 40, 62]. Specialist antenatal care for adolescents has not historically been associated with better objective outcomes than traditional antenatal care in other contexts [107]. However, components of this approach including continuity of care, speciality training for professionals and the emotional and social support gained through relationships with other adolescent services users, have been recognised as beneficial interventions in some HICs [107–109], and may be transferrable to LMICs. The varying contexts of LMICs mean no one single intervention can be recommended. Strategies need to be considered in the context of country specific opportunities and challenges. Creating opportunities for adolescents to be involved in care-planning and providing adolescents with choices, such as offering both routine antenatal care and adolescent specific services, could help meet adolescent specific needs.

This review had some limitations. Studies with a quality grading C, with flaws which may affect trustworthiness, were included in the synthesis. Although consistent with the approach of other authors undertaking qualitative synthesis [56–58], the lack of detail in the papers, meant methodological flaws which may affect the trustworthiness of findings could not be definitively excluded. Including English language papers only meant 12 papers were excluded which could have provided valuable insight, particularly from the countries in Latin America and the Caribbean. Only three low-income countries are represented in the review and few studies were from countries reporting the highest known incidence of adolescent pregnancy, reflecting the well cited recognition that research in low-resource settings remains a challenge [110–112], and that further research is needed. Male adolescents are not well represented. Research focused on their experiences would also be helpful in considering how to meet their needs. Finally, the ages of primary study participants were not reported by all authors. Based on the information provided, the youngest adolescents were not well represented. This could be due to poor reporting or because there are fewer pregnancies in this age range, but these adolescents are also least likely to have the agency to engage in research. Better understanding of their experiences would be beneficial considering this is the age of most significant change and development.

## Conclusions

Measures to reduce pregnancy incidence should remain a priority of the international community, particularly as many married adolescents do not yet want to be mothers. Adolescents who do experience pregnancy in LMICs want and need support to meet their personal and pregnancy related needs. Efforts must be made to increase the support available to adolescents through personal and professional relationships to allow this need to be met. Reducing the stigma of adolescent pregnancy, in regions where pregnancy remains unacceptable outside of marriage, is needed to improve the support available to adolescents in their personal relationships.

Increasing the availability of adolescent friendly and respectful care is integral to meeting the needs of adolescents who are pregnant. This requires more and better training of health care providers to have the knowledge and skills to provide respectful care to adolescents. Wherever possible adolescents should also be involved in the planning of health care services and efforts should be taken to offer traditional and adolescent focused services. Adolescents should then be supported to make decisions about their care. Creating opportunities for family members to collaborate on efforts to improve care provision could increase care-seeking among adolescents who have less control over decision-making and improve the family’s role as a source of support. The vast differences across countries mean policy makers and other stakeholders need to consider how these implications can be realised in each context.

## Supplementary Information

**Additional file 1:** **S1 - **Search strategy for each database. **S2** - Table created to display checklist authored by Walsh and Downe. **S3**- Summary of Quality Assessment of Studies.

## Data Availability

The datasets used and/or analysed during the current study available from the corresponding author on reasonable request.
