# Supplementary material for: Adolescent experiences of pregnancy in low-and middle-income countries: a meta-synthesis of qualitative studies
Source: BMC Pregnancy Childbirth. 2022 Sep 12;22:702. doi: 10.1186/s12884-022-05022-1 (PMC9469636; doi:10.1186/s12884-022-05022-1)
Supplement: Supplementary file 1 — Additional file 1: S1 - Search strategy for each database. S2 - Table created to display checklist authored by Walsh and Downe. S3- Summary of Quality Assessment of Studies. [file 12884_2022_5022_MOESM1_ESM.docx]

**Supplementary Information**

**S1 - Search strategy for each database**

- *Medline search terms (apply qualitative filter after combining searches)*

adolescen* (kw) OR teen*(kw) OR juvenile(kw) OR “young person”(kw) OR “young people”(kw) OR “young adult” (kw) OR youth (kw) OR “young mother” (kw) OR Adolescent (MeSH) OR Pregnancy in Adolescence (MeSH)

AND

pregnan* (kw) OR antenatal (kw) OR childbearing (kw) OR expectant (kw) OR prenatal (kw) OR Pregnancy (MeSH) OR Prenatal Care (MeSH)

AND

“developing countr*” (kw) OR “low to middle income” (kw) OR “low and middle income” (kw) OR LMIC (kw) OR “low resource” (kw) OR “low income” (kw) OR “middle income” (kw) OR “global south” (kw) OR “least developed countr*” (kw) OR “underdeveloped countr*” (kw) OR Developing Countries (MeSH)

- *CINAHL search terms (apply qualitative filter after combining searches)*

adolescen* (kw) OR teen*(kw) OR juvenile(kw) OR “young person”(kw) OR “young people”(kw) OR “young adult” (kw) OR youth (kw) OR “young mother” (kw) OR Adolescence (MeSH) OR “young adult” (MeSH) OR “adolescent mothers”

AND

pregnan* (kw) OR antenatal (kw) OR childbearing (kw) OR expectant (kw) OR prenatal (kw) OR pregnancy (MeSH) OR “prenatal care” (MeSH)

AND

“developing countr*” (kw) OR “low to middle income” (kw) OR “low and middle income” (kw) OR LMIC (kw) OR “low resource” (kw) OR “low income” (kw) OR “middle income” (kw) OR “global south” (kw) OR “least developed countr*” (kw) OR “underdeveloped countr*” (kw) OR Developing Countries (MeSH) OR “low and middle income” (MeSH)

- *Global Health search terms*

adolescen* (kw) OR teen*(kw) OR juvenile(kw) OR “young person”(kw) OR “young people”(kw) OR “young adult” (kw) OR youth (kw) OR “young mother” (kw) OR adolescents (The) OR adolescents OR “pregnant adolescents” OR youth (The)

AND

pregnan* (kw) OR antenatal (kw) OR childbearing (kw) OR expectant (kw) OR prenatal (kw) OR pregnancy (The) OR “prenatal period” (The) OR “Prepartum period” (The)

AND

“developing countr*” (kw) OR “low to middle income” (kw) OR “low and middle income” (kw) OR LMIC (kw) OR “low resource” (kw) OR “low income” (kw) OR “middle income” (kw) OR “global south” (kw) OR “least developed countr*” (kw) OR “underdeveloped countr*” (kw) OR “developing countries” (The) OR “low income” (The)

AND

Qualitative (kw) OR “Mixed methods” OR Qualitative analysis (The)

- *PubMed search terms*

adolescen* (kw) OR teen*(kw) OR juvenile(kw) OR “young person”(kw) OR “young people”(kw) OR “young adult” (kw) OR youth (kw) OR “young mother” (kw) OR adolescent (MeSH)

AND

pregnan* (kw) OR antenatal (kw) OR childbearing (kw) OR expectant (kw) OR prenatal (kw)

AND

“developing countr*” (kw) OR “low to middle income” (kw) OR “low and middle income” (kw) OR LMIC (kw) OR “low resource” (kw) OR “low income” (kw) OR “middle income” (kw) OR “global south” (kw) OR “least developed countr*” (kw) OR “underdeveloped countr*” (kw)

- *African Journals Online*

Adolescen* AND Experiences AND Pregnan* AND Qualitative

- *PsycINFO*

Adolescen* OR Teen* AND pregnan*OR childbirth AND qualitative

S2 - Table created to display checklist authored by Walsh and Downe

| **Stages** | **Essential Criteria** | **Specific Prompts** |
| --- | --- | --- |
| Scope and Purpose | Clear statement of, and  rationale for, research question/aims/purposes | - Clarity of focus demonstrated - Explicit purpose given, such as descriptive/explanatory intent, theory building, hypothesis testing - Link between research and existing knowledge demonstrated |
|  | Study thoroughly  contextualised by existing literature | - Evidence of systematic approach to literature review, location of literature to contextualise the findings |
| Design | Method/design apparent,  and consistent with research intent | - Rationale given for use of qualitative design - Discussion of epistemological/ontological grounding - Rationale explored for specific qualitative method (e.g., ethnography, grounded theory, phenomenology) - Discussion of why particular method chosen is most appropriate/sensitive/relevant for research question/aims - Setting appropriate |
|  | Data collection strategy apparent and appropriate | - Were data collection methods appropriate for type of data required and for specific qualitative method? - Were they likely to capture the complexity/diversity of experience and illuminate context in sufficient detail? - Was triangulation of data sources used if appropriate |
| Sampling strategy | Sample and sampling method appropriate | - Selection criteria detailed, and description of how sampling was undertaken - Justification for sampling strategy given - Thickness of description likely to be achieved from sampling - Any disparity between planned and actual sample |
| Analysis | Analytic approach appropriate | - Approach made explicit (e.g., Thematic distillation, constant comparative method, grounded theory) - Was it appropriate for the qualitative method chosen? - Was data managed by software package or by hand and why? - Discussion of how coding systems/conceptual frameworks evolved - How was context of data retained during analysis - Evidence that the subjective meanings of participants were portrayed - Evidence of more than one researcher involved in stages if appropriate to epistemological/theoretical stance - Did research participants have any involvement in analysis (e.g., member checking) - Evidence provided that data reached saturation or discussion/rationale if it did not - Evidence that deviant data was sought, or discussion/rationale if it was not |
| Interpretation | Context described and  taken account of interpretation | - Description of social/physical and interpersonal contexts of data collection - Evidence that researcher spent time ‘dwelling with the data’, interrogating it for competing/alternative explanations of phenomena |
|  | Clear audit trail given | - Sufficient discussion of research processes such that others can follow ‘decision trail’ |
|  | Data used to support  Interpretation | - Extensive use of field notes entries/verbatim interview quotes in discussion of findings - Clear exposition of how interpretation led to conclusions |
| Reflexivity | Researcher reflexivity demonstrated | - Discussion of relationship between researcher and participants during fieldwork - Demonstration of researcher’s influence on stages of research process - Evidence of self-awareness/insight - Documentation of effects of the research on researcher - Evidence of how problems/complications met were dealt with |
| Ethical Dimensions | Demonstration of  sensitivity to ethical  concerns | - Ethical committee approval granted - Clear commitment to integrity, honesty, transparency, equality and mutual respect in relationships with participants - Evidence of fair dealing with all research participants - Recording of dilemmas met and how resolved in relation to ethical issues - Documentation of how autonomy, consent, confidentiality, anonymity was managed |
| Relevance and Transferability | Relevance and  transferability evident | - Sufficient evidence for typicality specificity to be assessed - Analysis interwoven with existing theories and other relevant explanatory literature drawn from similar settings and studies - Discussion of how explanatory propositions/emergent theory may fit other contexts - Limitations/weaknesses of study clearly outlined - Clearly resonates with other knowledge and experience - Results/conclusions obviously supported by evidence - Interpretation plausible and ‘makes sense’ - Provides new insights and increases understanding - Significance for current policy and practice outlined - Assessment of value/empowerment for participants - Outlines further directions for investigation - Comment on whether aims/purposes of research were achieved |

S3 - Summary of Quality Assessment of Studies

| **Primary Author** | **Criteria** | | | | | | | | | | | | |
| --- | --- | --- | --- | --- | --- | --- | --- | --- | --- | --- | --- | --- | --- |
|  | Clear statement of, and rationale for, research question/aims/purposes | Study thoroughly  contextualised by existing | Method/design apparent,  and consistent with research intent | Data collection strategy  apparent and appropriate | Sample and sampling  method appropriate | Analytic approach  appropriate | Context described and taken account of in interpretation | Clear audit trail given | Quotes used | Researcher reflexivity demonstrated | Demonstration of sensitivity to ethical concerns | Relevance and transferability evident | Grade |
| AL-Kloub | Y | Y | Y | P | Y | Y | P | Y | Y | P | P | P | **B** |
| Apolot | Y | Y | P | Y | P | P | P | P | Y | N | Y | P | **C** |
| Asnong | Y | Y | P | Y | Y | Y | P | Y | Y | P | Y | Y | **B** |
| Astuti | Y | Y | Y | Y | Y | Y | P | P | Y | N | P | Y | **B** |
| Atuyambe (1) | Y | Y | Y | P | Y | P | P | Y | Y | N | N | P | **C** |
| Atuyambe (2) | Y | Y | Y | Y | P | P | Y | P | Y | N | Y | P | **B** |
| Bwalya | Y | Y | Y | P | Y | P | P | P | Y | P | Y | P | **C** |
| Chikalipo | Y | Y | Y | Y | Y | Y | P | P | Y | N | Y | Y | **B** |
| Duggan | Y | P | Y | Y | Y | Y | Y | Y | Y | P | Y | Y | **A** |
| Erasmus | Y | Y | Y | Y | P | Y | P | Y | Y | Y | Y | Y | **B** |
| Görgen | Y | Y | N | N | N | N | N | N | Y | N | P | P | **D** |
| Govender | Y | Y | P | P | P | Y | Y | Y | Y | Y | Y | P | **B** |
| Gyesaw | Y | Y | P | P | Y | P | P | P | Y | P | Y | P | **B** |
| James | Y | Y | Y | Y | P | N | Y | P | Y | P | Y | P | **C** |
| Mashala | Y | Y | Y | Y | P | N | P | P | Y | N | P | P | **C** |
| Mohammadi | Y | Y | Y | P | P | Y | Y | Y | Y | P | Y | P | **B** |
| Nabugoomu | Y | P | P | Y | Y | Y | P | P | Y | P | P | P | **B** |
| November | Y | Y | Y | Y | P | P | P | Y | Y | N | Y | P | **C** |
| Sewpaul | Y | Y | Y | Y | Y | P | Y | P | Y | P | Y | P | **B** |
| Shahabuddin (1) | Y | Y | P | Y | Y | P | Y | Y | Y | N | Y | P | **B** |
| Shahabuddin (2) | Y | Y | P | Y | P | P | Y | P | Y | N | Y | p | **C** |
| Tatum | y | Y | P | Y | P | N | P | P | Y | P | N | Y | **C** |
